# Unraveling the Role of Metabolic Endotoxemia in Accelerating Breast Tumor Progression

**DOI:** 10.3390/biomedicines13081868

**Published:** 2025-07-31

**Authors:** Daniela Nahmias Blank, Ofra Maimon, Esther Hermano, Emmy Drai, Ofer Chen, Aron Popovtzer, Tamar Peretz, Amichay Meirovitz, Michael Elkin

**Affiliations:** 1 Sharett Institute of Oncology, Hadassah-Hebrew University Medical Center, Jerusalem 91120, Israel; 2 Faculty of Medicine, The Hebrew University of Jerusalem, Jerusalem 91120, Israel; 3 Legacy Heritage Oncology Center and Dr. Larry Norton Institute, Soroka University Medical Center, Be’er Sheva 84101, Israel

**Keywords:** breast cancer, obesity, metabolic endotoxemia, inflammation, toll-like receptor

## Abstract

**Background:** Obese women have a significantly higher risk of bearing breast tumors that are resistant to therapies and are associated with poorer prognoses/treatment outcomes. Breast cancer-promoting action of obesity is often attributed to elevated levels of insulin, glucose, inflammatory mediators, and misbalanced estrogen production in adipose tissue under obese conditions. Metabolic endotoxemia, characterized by chronic presence of extremely low levels of bacterial endotoxin (lipopolysaccharide [LPS]) in the circulation, is a less explored obesity-associated factor. **Results:** Here, utilizing in vitro and in vivo models of breast carcinoma (BC), we report that subclinical levels of LPS typical for metabolic endotoxemia enhance the malignant phenotype of breast cancer cells and accelerate breast tumor progression. **Conclusions:** Our study, while focusing primarily on the direct effects of metabolic endotoxemia on breast tumor progression, also suggests that metabolic endotoxemia can contribute to obesity–breast cancer link. Thus, our findings add novel mechanistic insights into how obesity-associated metabolic changes, particularly metabolic endotoxemia, modulate the biological and clinical behavior of breast carcinoma. In turn, understanding of the mechanistic aspects underlying the association between obesity and breast cancer could help inform better strategies to reduce BC risk in an increasingly obese population and to suppress the breast cancer-promoting consequences of excess adiposity.

## 1. Introduction

The association between obesity and the increased risk/aggressive characteristics/therapy resistance of breast cancer (BC) is well documented [1,2,3]. While in postmenopausal patients obesity increases the risk of estrogen receptor-positive (ER+) BC subtypes, the incidence of triple-negative tumors (that do not express ER, progesterone receptor (PR), or human epidermal growth factor receptor 2 (HER2)) may be enhanced in premenopausal women with obesity [4,5,6,7]. Additionally, according to numerous reports, obese patients with BC have a worse disease-free and overall survival, compared to their normal-weight counterparts, despite appropriate local and systemic therapies (reviewed in [6]. Furthermore, obese BC patients experience more complications related to surgery, radiation, and chemotherapy, and are at increased risk for local recurrence compared to non-obese patients (reviewed in [3,6]).

BC-promoting action of obesity is undoubtedly multifactorial; among the factors contributing to BC progression under an obese state, most attention was attracted to increased circulating levels of insulin, glucose, inflammatory mediators, alterations in adipokine pathophysiology, and, in hormone-responsive BC subtypes, misbalanced estrogen production in adipose tissue [6]. Less explored is the connection between BC and metabolic endotoxemia (ME). ME is a condition characterized by the chronic presence of extremely low (typically 0.1 to 1 EU/mL), but biologically significant levels of bacterial endotoxin in the plasma of obese patients and experimental models of obesity [8,9,10,11,12,13,14,15,16].

ME occurs in the face of a change in gut microbiota composition, due to impaired intestinal permeability under obese conditions, leading to the translocation of small quantities of endotoxin from the gut lumen to the circulation [8,17,18,19]. Numerous studies implicated ME in pathogenesis of several obesity-associated pathophysiological conditions (reviewed in [8]). However, the link between ME and BC was not previously investigated.

Endotoxin is a potent trigger of inflammatory responses, acting through activation of toll-like receptor 4 (TLR4) signaling. TLR4 is well-known for its role in innate immunity [20]. It is broadly represented on immunocytes (especially on antigen-presenting cells) and involved in the recognition of molecular patterns associated with bacterial pathogens, i.e., endotoxin. However, TLR4 is expressed by the epithelial cells as well, including those originating from BC (Figure S1 and references [21,22,23,24,25]). On the one hand, in cancer patients, TLR4 (expressed by immunocytes) can mediate activation of innate or adaptive immunity, and could confer benefit, as several reports indicate the potency of TLR agonists in enhancing antitumor immune responses [26]. On the other hand, it was suggested that endotoxin-triggered activation of TLR4 found on carcinoma cells may contribute to cancer progression/resistance to therapy in tumors of various anatomic sites [27,28,29]. Moreover, analyzing the association between TLR4 expression in tumor biopsies and overall survival in a cohort of 1247 breast carcinoma patients (retrieved from a publicly available dataset https://xena.ucsc.edu, accessed on 15 February 2023), we found that expression of TLR4 was associated with significantly lower overall survival (Figure S2). Interestingly, levels of TLR4 expression in BC cells of human and mouse origin are comparable with those detected in immunocytes (Figure S1B). Incorporating these observations with the occurrence of ME under obese conditions, along with the accelerated BC tumor progression in diet-induced obesity mouse model associated with ME (Figure S3), we hypothesized that chronically elevated levels of endotoxin in BC patients with excess adiposity may sustain obesity-related breast tumor progression.

In this study, utilizing in vitro and in vivo models of BC, we investigated whether ME contributes to accelerated BC progression and, if so, by what mechanism. We found that presence of LPS at subclinical levels, typical for ME, enhances the malignant phenotype of TLR4-expressing BC cells, and in parallel, adversely activates host-derived cells infiltrating the tumor tissue. Given the numerous reports linking ME to the obese state (both in clinical and experimental settings) [8,9,10,11,13,14,15,16], our study suggests potential links between metabolic endotoxemia and breast tumorigenesis, contributing to the evolving understanding of how obesity-related changes modulate breast cancer behavior.

## 2. Materials and Methods

### 2.1. Cell Culture

Human BC cell lines MDA-MB-231 (ER-negative) and MCF7 (ER-positive) were authenticated by short tandem repeat profiling at the Genomics Center of the Biomedical Core Facility, Technion University, Haifa, Israel, and cultured in DMEM; human promonocytic cell line THP-1 and murine BC cell line E0771 kindly provided by Dr. R. Sharon, Hebrew University Medical School (Jerusalem, Israel) were grown in RPMI 1640 medium, supplemented with 1 mM glutamine, 50 μg/mL streptomycin, 50 U/mL penicillin, and 10% fetal calf serum (Biological Industries, Beit Haemek, Israel) at 37 °C and 8% CO_2_. Some cells were treated with endotoxin (LPS, 0.1 ng/mL). Ultrapure LPS from *E coli* 0111:B4 (Invitrogen, Waltham, MA, USA) was used, to ensure absence of contaminating TLR agonists (i.e., lipoproteins).

### 2.2. Murine Model of Metabolic Endotoxemia-Associated BC

Mouse model of metabolic endotoxemia was established essentially as described [13]. Briefly, nude or syngeneic C57BL/6 mice were implanted subcutaneously with the micro-osmotic pumps (Alzet, Campbell, CA, USA; Model 1004), filled with saline (control) or with LPS to infuse 300 μg × kg^−1^ × Day^−1^ (these conditions produce plasma LPS concentrations similar to those found in ME [13]). Three days after pump implantation, when the LPS infusion rate reaches a constant level and remains stable for 28 days [30], MDA-MB-231 or E0771 cells (5 × 10^5^ cells per injection) were injected subcutaneously or orthotopically into the mammary fat pad of nude or C57BL/6 mice, respectively. To confirm the occurrence of experimental ME, circulating levels of LPS were measured 14 days post-pump implantation, as described below. Tumor growth was monitored for 14 (MDA-MB-231) or 15 (E0771) days and mice were then sacrificed. Intratumoral bacterial LPS was detected immunohistochemically on formalin-fixed paraffin-embedded tumor tissue, using a monoclonal mouse anti-LPS antibody detecting the core bacterial LPS antigen (clone WN1 222-5, Hycult Biotech, Uden, The Netherlands) at a 1:800 dilution.

### 2.3. Chromogenic Kinetic Limulus Amoebocyte Lysate Assay (LAL Test)

Blood samples derived from obese/lean BC patients and from mice implanted with saline/LPS-infusing pump were collected into heparin-coated tubes. Plasma was separated by centrifugation at 4 °C and kept frozen at −20 °C. Endotoxin determination was performed with the chromogenic kinetic Limulus Amoebocyte Lysate assay for the detection of Gram negative bacterial endotoxin applying Endosafe-nextgen portable test system apparatus and multi-cartridge system readers (Charles River Endosafe, Charleston, SC, USA). The cartridges used were certified to enable a lower limit of detection of 0.005 EU/mL endotoxin. Plasma samples were diluted 1:10, heat inactivated for 15 min at 70° C to minimize protease interference and further diluted 1:10 with Endosafe LAL reagent water and assayed in duplicates.

### 2.4. MTS Assay

Cells were seeded in 96-well culture plates in serum-free DMEM/RPMI. MTS assay (Promega, Madison, WI, USA) was performed according to the manufacturer instructions and cell proliferation was measured after addition of 0.1 ng/mL LPS/0.25 μM Doxorubicin. Each experiment was performed at least three times. Each data point shows the mean of pentaplicate cultures.

### 2.5. Immunoblotting

E0771, MDA-MB-231 and MCF7 cells, untreated or treated with 0.1 ng/mL LPS, or snap-frozen tumor tissue samples were homogenized in lysis buffer containing 0.6% SDS, 10 mM Tris-HCl, pH 7.5, supplemented with a mixture of protease inhibitors (Roche, Basel, Switzerland) and phosphatase inhibitors (Thermo Scientific, Waltham, MA, USA). Equal protein aliquots were subjected to SDS-PAGE (10% acrylamide) under reducing conditions and proteins were transferred to a polyvinylidene difluoride membrane (Millipore, Burlington, MA, USA). Membranes were blocked with 3% BSA for 1 h at room temperature and probed with the appropriate antibody, followed by horseradish peroxidase-conjugated secondary antibody (Thermo Scientific, Waltham, MA, USA) and a chemiluminescent substrate (GeBA, Yavne, Israel). Band intensity was quantified by densitometry analysis using ImageJ software (version 1.54).

### 2.6. Analysis of Gene Expression by Quantitative Real-Time PCR Analysis (qRT-PCR)

Total RNA was isolated from whole cells, untreated or treated with 0.1 ng/mL LPS at different time points in vitro and from snap-frozen tumor tissue samples using TRIzol (Invitrogen, Waltham, MA, USA), according to the manufacturer’s instructions, and quantified by spectrophotometry. Single-stranded cDNA was amplified from 1 μg of total RNA using qScript cDNA Synthesis Kit (Quanta Bio, Beverly, MA, USA). Real-time quantitative PCR (qRT-PCR) analysis was performed with an automated rotor gene system RG-3000A (Corbett Research, Hilden, Germany). The PCR reaction mix (20 µL) was composed of 10 µL QPCR sybr master mix (Quanta Bio, Beverly, MA, USA), 5 µL of diluted cDNA (each sample in triplicate), and a final concentration of 0.3 µM of each primer. Hypoxanthine guanine phosphoribosyl transferase (HPRT) primers were used as an internal standard. The primers utilized in the study are listed in Table S1.

### 2.7. Antibodies

Immunoblotting, immunostaining, and immunofluorescence were carried out with the following antibodies: anti-TLR4 (ab13556 Abcam, Cambridge, UK MAB2759 R&D Minneapolis, MN, USA), anti-phospho-STAT3 (Tyr705) (D3A7 Cell Signaling, Danvers, MA, USA) anti–STAT3 (H-190) (sc-7179, sc-8019 Santa Cruz, Dallas, TX, USA), anti-phospho-AKT (Ser4730) (9271 Cell Signaling, Danvers, MA, USA), anti–AKT (9272 Cell Signaling, Danvers, MA, USA), anti-phospho-ERK1/2 (Thr202) (4370 Cell Signaling, Danvers, MA, USA), anti-ERK1 (K-23) (sc-94 Santa Cruz, Dallas, TX, USA), anti-Actin (C-2) (sc-8432 Santa Cruz, Dallas, TX, USA), anti-GAPDH (V-18) (sc-20357 Santa Cruz, Dallas, TX, USA), anti-Ki67 (SP6 Thermo Scientific, Waltham, MA, USA), anti–αSMA (1A4 DAKO, Glostrup, Denemark), anti-F4/80 (MCA497R SEROTEC, Kidlington, UK), anti-LPS (HM6011 Hycult biotech, Uden, The Netherlands), and anti–IL-6 (Santa Cruz, Dallas, TX, USA).

### 2.8. Immunofluorescence

For immunofluorescence analysis, DyLight 488 goat anti-rat and CyTM3 donkey anti-rabbit (The Jackson Laboratory, Bar Harbor, ME, USA) antibodies were used as secondary antibodies. Nuclear staining was performed with 4′,6-diamidino-2-phenylindole (DAPI) or 1,5-bis{[2-(di-methylamino)ethyl]amino}-4,8-dihydroxyanthracene-9,10-dione (DRAQ5) (Cell Signaling Technology, Danvers, MA, USA). Images were captured using a Zeiss LSM 5 confocal microscope and analyzed with Zen software (Carl Zeiss, Oberkochen, Germany) and ImageJ software.

### 2.9. Immunohistochemistry

Paraffin-embedded slides were deparaffinized and incubated in 3% H_2_O_2_. Antigen retrieval was carried out by heating (20 min) in a microwave oven in 10 mM Tris buffer containing 1 mM EDTA (for αSMA staining) or by citrate buffer (for Ki67 and LPS staining) or by treatment (5 min) with Pronase (for F4/80 staining). Slides were incubated with primary antibodies diluted in CAS-Block (Invitrogen) or with CAS-Block alone, as a control. Appropriate secondary antibodies (Nichirei, Tokyo, Japan) were then added and slides were incubated at room temperature for 30 min. Mousestain kit (Nichirei, Tokyo, Japan) was used when primary mouse antibodies were applied to stain mouse tissues. Color was developed using the DAB substrate kit (Thermo Scientific, Waltham, MA, USA), followed by counter staining with Mayer’s hematoxylin. Staining with the control IgG or without addition of primary antibody showed low or no background staining in all cases.

### 2.10. Migration and Invasion Assay

To assess cell motility and invasiveness, 0.6 × 10^5^ cells/mL (for migration) and 1 × 10^6^ cells/mL (for invasion) were seeded on polycarbonate membrane inserts with 8 µm pore size. CytoSelect 24-Well Cell Fluorometric Migration and CytoSelect 24-Well Cell Colorimetric Invasion assays (Cell BioLabs, San Diego, CA, USA) were performed according the manufacturer’s instructions. Briefly, cell suspension was placed in the upper chamber of inserts in DMEM medium without serum overnight. The day after, supernatants in the upper chamber were replaced with medium containing 0.1 ng/mL LPS or control medium for 24 h for migration assay and 48 h for invasion assay. Migratory cells pass through basement membrane layer to the bottom chamber of the insert. For migration, cells were dissociated from the membrane and detected in a fluorescence reader using the CyQuant^®^ GR Dye (Invitrogen, Waltham, MA, USA). For invasion, basement membrane-coated inserts were used, non-invasive cells (upper chamber) were removed, and the inserts were incubated with cell stain solution for 10 min at RT. After that, cells were washed several times with sterile water and allowed to dry. Finally, extraction solution was added for 10 min incubation. Absorbance was read with a spectrophotometer at 560 nm.

### 2.11. Statistical Analysis

Significance of differences between the means was analyzed using the unpaired Student’s *t*-test or, when the requirement of normal distribution for the *t*-test was not met, the Mann–Whitney test. All statistical tests were two-sided. A *p* value < 0.05 was considered statistically significant.

### 2.12. Study Approval

Animal experiments were approved by the Institutional Animal Care and Use Committee of the Hebrew University.

## 3. Results

### 3.1. ME Conditions Lead to Activation of Key BC-Promoting Pathways and Stimulate BC Cell Growth/Chemo-Resistance

**Figure 1:**
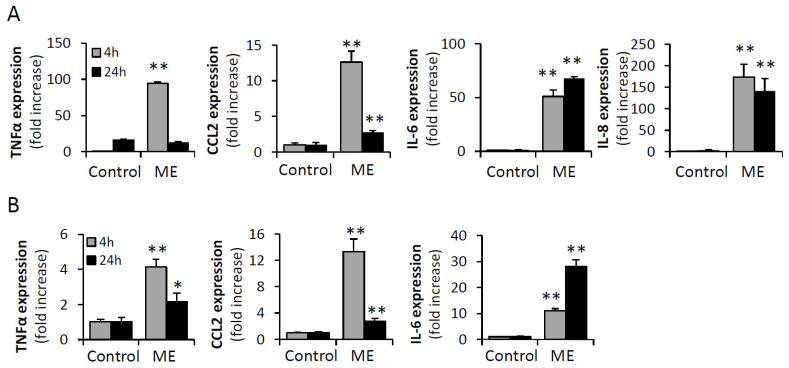
LPS at concentrations reflecting metabolic endotoxemia induces tumor-promoting cytokines in BC cells. Human MDA-MB-231 (**A**) and mouse E0771 (**B**) BC cells were serum-starved overnight and then either remained untreated (control) or incubated with LPS (0.1 ng/mL, mimicking metabolic endotoxemia conditions, ME) for 4 h (grey bars) and 24 h (black bars). Expression of TNFα, IL-6, CCL2, and human IL-8 was analyzed by qRT-PCR. Error bars, ±SE. Student’s *t*-test * *p* < 0.02; ** *p* < 0.01.

Previous reports have suggested tumor-promoting effects of endotoxin-elicited TLR4 signaling in “sterile” cancer types (including BC) [22,24,27,28,29]. Nevertheless, current studies on endotoxin-mediated responses in cancer cells were performed using exceedingly high concentrations of endotoxin (>10 ng/mL), which surpass at least by an order of magnitude the ME levels reported in clinical and experimental obesity [8,11,12,13,14,15,16] and, given extraordinary sensitivity of humans to endotoxin [31], is sufficient to cause septic shock [32,33]. Thus, to validate functionality of endotoxin-TLR4 signaling axis under ME conditions, we incubated human (MDA-MB-231) and murine (E0771) BC cells (expressing TLR4 receptor at levels comparable to those detected in human/murine cells of monocytic origin (Figure S1B)), with endotoxin (LPS, 0.1 ng/mL), reflecting levels typically observed in obese patients and in experimental ME [8,11,12,13,14,15,18]. As BC cells are highly secretory, and their secretory activity could be modulated by inflammatory stimuli present in the tumor microenvironment [34], we first examined changes in cytokine expression. As shown in Figure 1, under ME conditions in vitro, we detected in BC cells significantly increased expression of TNFα, CCL2, IL-6 and human IL-8, well-known TLR-controlled cytokines, tightly implicated in breast tumorigenesis [35,36,37,38,39,40,41] and the obesity-cancer link [6,42,43,44,45]. Given previous reports, showing increased TLR4 expression in response to LPS in several cell types other than BC [46,47], we examined whether similar upregulation occurs in BC cells. We found that ME conditions resulted in twofold increase of TLR4 mRNA expression in MDA-MB-231 cells (±0.25, *p* value < 0.001) and 1.3 fold increase in E0771 (however, in this cell line the increase did not reach statistical significance). These findings further support activation of endotoxin-TLR4 signaling axis under ME conditions.

**Figure 2:**
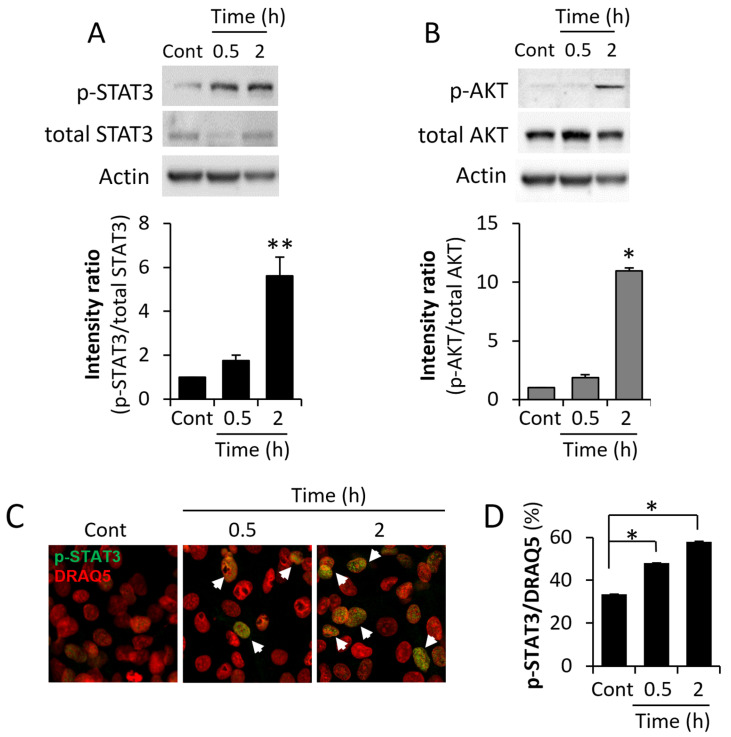
Activation of STAT-3 and AKT signaling pathways in BC cells under ME conditions in vitro. (**A**,**B**) MDA-MB-231 cells were serum-starved overnight and then either remained untreated (Cont) or incubated with LPS (0.1 ng/mL, mimicking ME conditions) for 0.5 h and 2 h. At the indicated time points, cells were harvested, and cell lysates containing equivalent amounts of total protein were immunoblotted using antibody specific for phospho-STAT3 (p-STAT3), phospho-AKT (p-AKT), total STAT3, total AKT, or total Actin (the Actin bands presented in panels A and B are from the same membrane). Band intensity was quantified using ImageJ software and intensity ratio (phospho/total) was calculated. The data shown are representative of three independent experiments. Mann–Whitney ** *p* < 0.01; Student’s *t*-test * *p* < 0.05. (**C**) MDA-MB-231 cells were seeded on coverslips in quadruplicates and untreated (Cont) or stimulated with LPS (0.1 ng/mL, mimicking ME conditions) for 0.5 h and 2 h. The cells were then stained with anti-pSTAT3 antibody (green). Cell nuclei were counterstained with DRAQ5 (red). Overlay (orange, indicated by white arrowheads) represents cells positive for nuclear-localized p-STAT3. (**D**) Quantification of the degree of association between p-STAT3 and DRAQ5 staining within MDA-MB-231 cells was performed using colocalization tool of ImageJ software, microscopic field = 0.0076 mm^2^; ≥100 cells per coverslip were analyzed, four coverslips/condition. Error bars, ±SE. Student’s *t*-test * *p* < 0.05.

**Figure 3:**
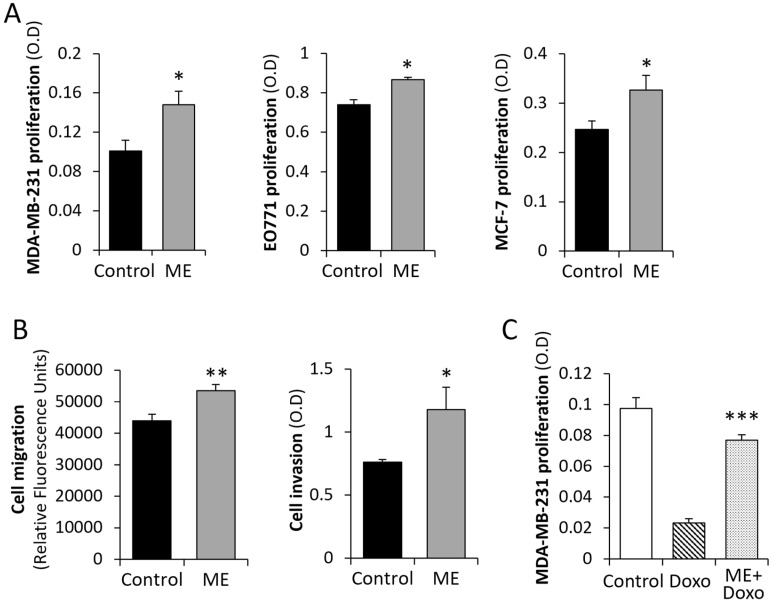
Pro-cancerous effects of ME conditions on cultured BC cells. (**A**) ME stimulates proliferation of human and mouse BC cells in vitro. MDA-MB-231 (left), E0771 (middle), and MCF-7 (right) cells were plated on 96-well plates (in quadruplicates) and cultured for 3 days either alone (control, black bars) or under conditions mimicking ME state (0.1 ng/mL of LPS, grey bars). Cell numbers were determined applying MTS proliferation assay. Data are the mean ± SD; Student’s *t*-test * *p* < 0.01. (**B**) Increased migration and invasion in vitro under ME conditions. Migration (left) and invasion (right) properties of MDA-MB-231 cells were assessed in the absence or presence of 0.1 ng/mL LPS, using CytoSelect Migration and Invasion assays, as described in Methods. Data are the mean ± SD; Student’s *t*-test. * *p* < 0.02 ** *p* < 0.01**.** (**C**) ME conditions render BC cells resistant to Doxorubicin. MDA-MB-231 cells were plated on 96-well plates (in pentaplicates) and cultured either alone (Control, empty bar) or in the presence of Doxorubicin (Doxo, 0.25 μM, striped bar) for 72 h. To some wells LPS (0.1 ng/mL) was added 3 h prior to Doxo, to mimic metabolic endotoxemia (ME) conditions (ME + Doxo, dotted bar). Cell growth was analyzed by MTS Cell Proliferation Assay. Data are the mean ± SD. Student’s *t*-test *** *p* < 0.01.

We next evaluated effect of ME conditions in vitro on activation of key cancer-promoting signal transduction pathways implicated in crosstalk with TLR4 signaling cascade (reviewed in [48,49]) as well as in breast tumorigenesis, and known to be triggered by the aforementioned cytokines [6,50], i.e., STAT3 and AKT. As shown in Figure 2, we found that ME conditions in vitro resulted in marked augmentation of signaling along STAT3 and AKT pathways, reflected by increased levels of phosphorylated STAT3 and AKT (Figure 2A,B), as well as augmented nuclear localization of pSTAT3 (Figure 2C,D) in MDA-MB-231 cells treated with 0.1 ng/mL of LPS. Similar increase in levels of pSTAT3 and pAKT was observed in murine E0771 and human MCF-7 (pAKT only) BC cells (Figure S4). In agreement with the detected enhancement of AKT and STAT3 signaling, we found that ME conditions stimulated in vitro proliferation of BC cells of human (MDA-MB-231, MCF-7) and mouse (E0771) origin (Figure 3A). In a similar way, ME conditions increased invasion (and to a lesser extent–migration) potential of BC cells in vitro (Figure 3B and Figure S5, left, middle panels). Additionally, ME conditions attenuated cytotoxic effect of Doxorubicin (backbone of chemotherapy treatment in BC) on MDA-MB-231 (Figure 3C), E0771 and MCF-7 (Figure S5, right panels) cells. These observations, taken together with the enhanced activation of STAT3 and AKT signaling shown in Figure 2, aligns with growing evidence that LPS-mediated TLR4 activation promotes chemo-resistance in cancer [51,52] by engaging pro-survival and anti-apoptotic pathways (particularly the AKT/NF-κB/STAT3 cascades) [51,53], thereby diminishing the cytotoxic effectiveness of chemotherapeutic agents such as doxorubicin.

### 3.2. Experimental ME Accelerates Breast Tumor Progression in Vivo

**Figure 4:**
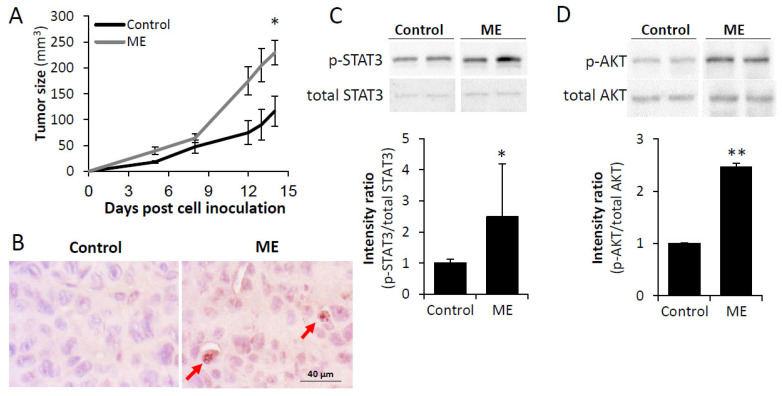
Chronic experimental metabolic endotoxemia accelerates BC progression in vivo. (**A**) Nude mice were implanted sc. with the Alzet osmotic minipump (as described in Methods) filled with either saline (Control) or LPS (to infuse 300 μg per kg per day, resulting in plasma LPS concentrations corresponding to metabolic endotoxemia, ME). Human BC cells MDA-MB-231 were injected 3 days after pump implantation, as described in Methods. Tumor growth was monitored for 14 days, *n* ≥ 3 mice per condition. (**B**) Immunohistochemical staining with anti-LPS antibody (as described in Methods) detected intratumoral LPS in tissue sections of tumors derived from LPS-infused, but not control (saline-infused) mice. Original magnification ×400. Scale bars indicate 40 µm. (**C,D**) Chronic experimental metabolic endotoxemia activates tumor-promoting signaling pathways STAT3 (**C**) and AKT (**D**). Tumor tissue lysates containing equivalent amounts of total protein were immunoblotted using antibody specific for phospho-STAT3 (p-STAT3), total STAT3 (**C**), phospho-AKT (p-AKT), and total AKT (**D**). Band splicing is indicated by spaces. Band intensity was quantified using ImageJ software and intensity ratio (phospho/total) was calculated. Data are the mean ± SD; Student’s *t*-test * *p* < 0.04; ** *p* < 0.01.

**Figure 5:**
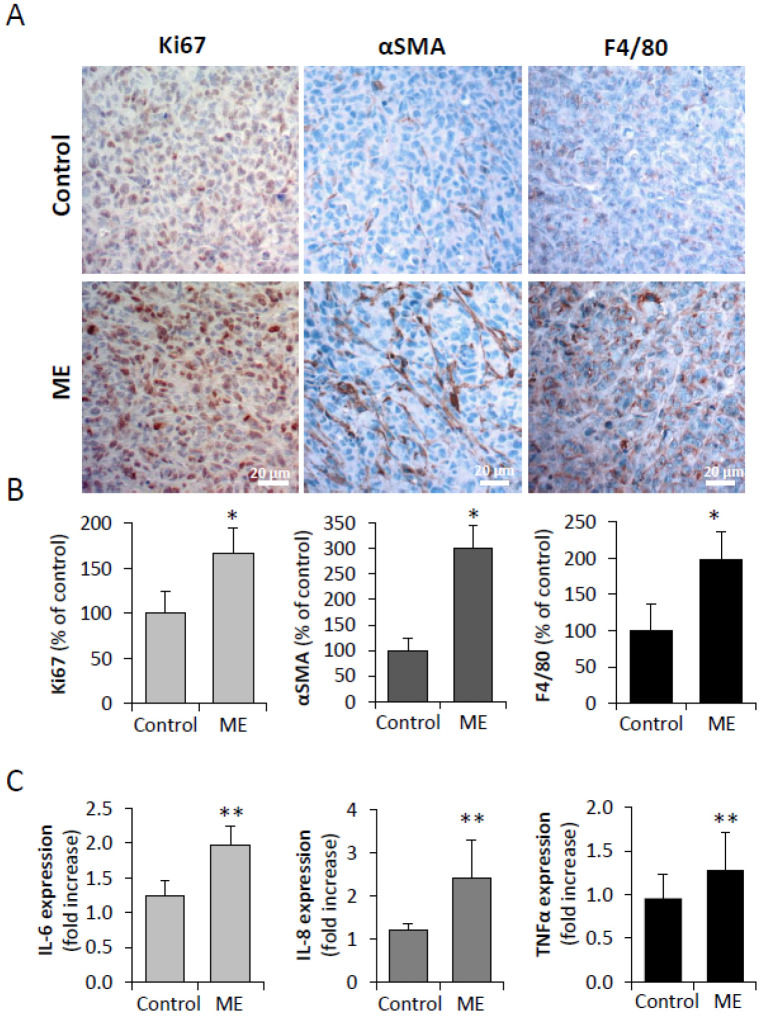
Effect of chronic experimental ME on stromal activation in BC tumors in vivo. (**A**) Representative photographs of tumor tissue sections derived from mice implanted with the Alzet minipumps filled with saline (control) or LPS (ME) as detailed in Methods, were immunostained for Ki67 (left), smooth muscle actin (αSMA-middle) and F4/80 (right). (**B**) Quantification per 0.0035 mm^2^ (Ki67 and F4/80) or 0.0022 mm^2^ (αSMA) microscopic fields (all the fields chosen for analysis were located >1 mm from the tumor border), based on at least three sections from three mice per group. (**C)** Expression of BC-promoting cytokines (human IL-6, IL-8, TNFα) in tumor tissue samples was analyzed by qRT-PCR. Error bars, ±SE. Student’s *t*-test. * *p* < 0.01, ** *p* < 0.04.

To validate the above findings in the context of ME-associated BC progression in vivo, we have established a mouse-based experimental system combining transplantable breast tumor xenografts (human MDA-MB-231 and murine E0771) with a murine model of chronic experimental ME [13], in which LPS-loaded osmotic mini-pump is implanted subcutaneously, sufficient to perform a chronic continuous infusion of LPS for 28 days, as described in Methods. The LPS infusion rate reaches a constant level 2 days after pump implantation, in accordance with the manufacturer’s data [30]. Experimental ME was further confirmed by detection of LPS [as described in Methods, (0.15 EU/mL, i.e., levels comparable with clinically-relevant endotoxin concentrations typical for ME) [10,11,12,13,14,15,16], in plasma of experimental (LPS-infused) mice 14 days post pump implantation. No detectable levels of LPS were identified in the plasma of control mice, implanted with saline-loaded pumps. Three days after pump implantation, MDA-MB-231 cells were injected subcutaneously in experimental and control nude mice and tumor growth was monitored for 14 days (Figure 4A). Then mice were sacrificed and their tumors excised. Confirming the experimental ME conditions, immunohistochemical analysis detected intratumoral LPS in tissue sections of tumors derived from LPS-infused, but not from saline-infused mice (Figure 4B). Chronic experimental ME markedly accelerated MDA-MB-231 tumor progression: twofold larger tumors were detected in LPS-infused vs. saline-infused mice 14 days post-tumor inoculation (Figure 4A). Applying qRT-PCR, we also detected increase expression of the cytokines IL-6, IL-8, and TNFα, key players in the obesity-associated BC progression [6] in tumors grown under ME conditions (Figure 5C). Of note, utilization of PCR primers specific for human IL-6, IL-8, and TNFα allowed to demonstrate that ME-induced expression of the cytokines occurred in BC cells per se, in agreement with the in vitro results (Figure 1). In further agreement with the in vitro findings (Figure 2), experimental ME also resulted in activation of STAT3 and AKT signaling pathways in MDA-MB-231 tumors in vivo (Figure 4C,D). Similarly to human MDA-MB-231 tumor progression, ME accelerated growth of murine E0771 BC tumors, which were injected orthotopically into syngeneic C57BL/6J mice pre-implanted with either LPS- or saline-loaded mini-pumps (Figure S6). Notably, comparable acceleration of the E0771 BC growth was observed in the tumors growing in obese (high fat diet-fed) vs. lean (control diet-fed) C57BL/6J mice (Figure S3). As high-fat diet-fed induced obesity in C57BL/6J mice is accompanied by development of ME [13,54], altogether these observations suggest involvement of ME in obesity-related acceleration of BC.

We next applied immunohistochemical analysis to examine the effects of ME on proliferation index and reactivation of the BC tumor stroma (hallmark of enhanced malignant phenotype in BC [55,56,57]). As shown in Figure 5A,B, immunostaining with antibody directed against proliferation marker Ki-67 revealed a highly significant increase in the rate of BC cell proliferation in the MDA-MB-231 tumors growing under experimental ME conditions, in agreement with the greater tumor size detected in mice implanted with the endotoxin-infusing pumps (Figure 4A).

**Figure 6:**
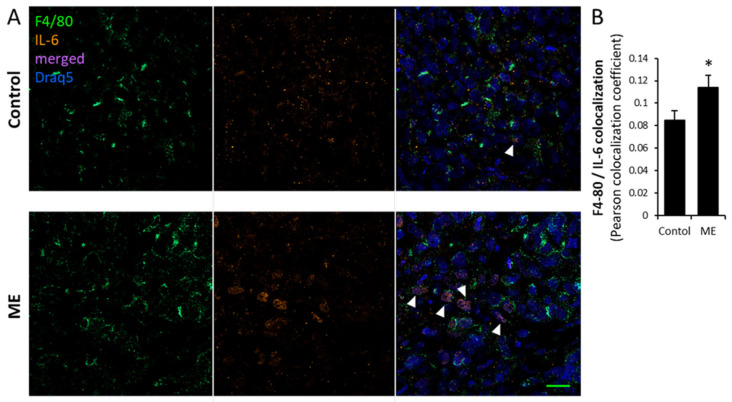
Increased levels of IL-6 expressing macrophage infiltration in BC tumor tissue are associated with metabolic endotoxemia in vivo. (**A**) MDA-MB-231 cells were injected sc. in nude mice implanted sc. with the Alzet osmotic minipump filled with either saline (control) or LPS (ME). Representative photographs of tumor tissue sections processed for colocalization immunofluorescent analysis using anti-IL-6 (orange) and anti-F4/80 (macrophage-specific marker, green) antibodies, as described in Methods. Cell nuclei were counterstained with DRAQ5 (blue). Overlay (pink, arrowheads) represents IL-6 positive-F4/80 positive macrophages. Scale bar: 20 µm. (**B**) Quantification of the degree of association between IL-6 and F4/80 staining within tumor tissue was performed using colocalization tool of Zen software (Carl Zeiss). Bar graph shows the Pearson’s correlation coefficient values for IL-6/F4/80 colocalization, * *p* < 0.04.

As metabolic deregulation and obesity are known to promote breast tumorigenesis through adverse activation of key cellular elements in tumor stroma, i.e., cancer associated fibroblasts, (CAF) and tumor-associated macrophages (TAM) [6], both expressing TLR4 [58,59], we next examined effects of experimental ME on the stromal compartment of the tumors. Immunostaining with antibody directed against alpha smooth muscle actin (αSMA, marker of activated CAFs) detected relatively modest levels of activated fibroblasts in tissue sections of tumors derived from saline-infused mice (Figure 5A). By contrast, experimental ME led to a marked increase in activated αSMA-positive fibroblasts in the tumor tissue of endotoxin-infused mice (Figure 5A,B). Additionally, applying immunostaining with antibodies directed against mouse macrophage markers F4/80 (Figure 5A,B; Figure 6), we observed significantly augmented infiltration of TAM in the tumors grown under ME conditions. As stated above, reactivation of tumor stroma, manifested by increased numbers of αSMA-positive CAF and TAM in tumors grown under ME conditions, is a hallmark of BC progression in general [57], and tightly implicated in as an obesity–BC link [6]. In particular, along with the carcinoma cell per se, TAM represent an important cellular source of several pro-cancerous cytokines, i.e., IL-6, postulated to fuel obesity-associated breast tumorigenesis [6,45]. In agreement with this notion, colocalization experiments revealed a statistically significant increase in the percentage of IL-6 positive macrophages infiltrating tumors growing in endotoxin-infused vs. control mice (Figure 6), further supporting contribution of ME conditions to BC stroma reactivation. Given the well-documented capacity of LPS to directly activate fibroblasts and macrophages [58,60,61,62], it is plausible that the stromal reactivation observed here results, at least in part, from a direct effect of metabolic endotoxemia. However, cytokines induced by ME in breast carcinoma cells themselves (Figure 1), notably CCL2—a key chemokine mediating macrophage recruitment in obesity-associated breast tumors [42]—may also play a role in driving this stromal activation.

## 4. Discussion

Microbes and microbial products are believed to affect cancer development and therapy response at a variety of body sites [63,64], including the breasts [65,66,67,68,69,70]. LPS is a microbial component abundant in the human gut and the modern diet [71]. Under metabolic deregulation, small quantities of LPS, the canonical ligand of TLR4, are translocated from the gut lumen to the circulation. Indeed, elevated plasma LPS concentrations (i.e., ME) were documented in obese patients and in animal models of obesity [8,11,12,13,14,15,17]. Furthermore, ME was implicated in pathogenesis of several clinical and experimental obesity-associated non-malignant disorders, i.e., type 2 diabetes, non-alcoholic fatty liver disease, pancreatitis, atherothrombosis, and cardiovascular disease [13,14,15,72,73]. Recent reports have also linked metabolic endotoxemia (ME) to the pathogenesis of tumors other than BC [48,74]. However, contribution of ME to obesity–BC link was not investigated. Most attention was attracted to the contribution of adipose-tissue-derived estrogen to obesity-related progression of the estrogen receptor (ER) positive BC subtypes [75]. The impact of an obese state on ER-negative BC is less unequivocal; nevertheless, numerous studies support the estrogen-independent tumor-promoting effects of obesity as well [4,6,7,76,77]. Our present study attests ME as a new player in breast tumorigenesis. Moreover, given occurrence of ME under obese conditions, together with observations that BC cells express functional TLR4 and that TLR4 expression is associated with decreased survival of the BC patients (Figure 1 and Figure 2), it is conceivable that chronically increased levels of LPS may contribute to obesity-associated BC.

Worth mentioning, the exact role of TLR4 ligands in tumor progression has not been fully clarified [23]. On the one hand, in cancer patients signaling through TLR (expressed by immunocytes) can mediate activation of innate or adaptive immunity, and could confer benefit, as several reports indicate the potency of TLR agonists in enhancing antitumor immune responses [26]. On the other hand, recent data also suggest that signaling along endotoxin-TLR4 axis contributes to progression of several cancer types. Moreover, this contribution is relevant not only in tumors developing in the presence of abundant commensal flora (e.g., colorectal cancer [78]) but also in “sterile” cancer types at several anatomic sites, including breast [21,22,23,25,79]. Important to note, however, that in previous studies on tumor-promoting effects of endotoxin-TLR4 signaling, endotoxin was utilized at concentrations known to cause septic shock [31,32,33] and surpass by an order of magnitudes the levels that could be reached at setting of “sterile” tumors or reported in ME [8,11,12,13,15].

Thus, to address the paucity of experimental data on effects of ME in obesity-influenced breast tumorigenesis, we established an in vivo model of chronic ME-associated BC, properly reflecting LPS concentrations found under obese conditions. Here we show that chronically-elevated circulating levels of endotoxin, typical for clinical ME, accelerates breast tumor progression in the mouse host. Of note, utilization of ER-negative MDA-MB-231 cells in our experiments allowed us to specifically investigate the hormone-independent, direct effects of ME on BC progression, independent of estrogen–ER signaling. This distinction is important because, in ER-positive breast cancer, ME may additionally promote tumor growth indirectly through activation of adipose- and tumor-resident macrophages, which can enhance local estrogen production and thereby stimulate ER signaling pathways [62]. In this respect, it is worthy to note that when immunocompetent C57/BL6 female mice and syngeneic ER+ BC cells (E0771) were utilized in the above-described in vivo model, experimental ME accelerated tumor growth to even a higher degree (Figure S6), consistent with previous reported ability of obesogenic milieu components (including endotoxin at ME-relevant level) to upregulate ER-mediated responses in the setting of obesity-associated BC [80,81].

In vitro, we found that endotoxin at extremely low concentration, reflecting ME conditions, profoundly affect biological behavior of human and murine BC cells, leading to upregulation of key pro-cancerous cytokines and activation of chief BC-promoting signaling pathways, thus stimulating BC cell proliferation and aggressive behavior (i.e., migratory ability, invasiveness and chemo-resistance). A limitation of our study is that ME-induced cytokine secretion/TLR-4 levels were not assessed at the protein level; future studies incorporating protein-level analyses, would provide important complementary data. Moreover, although the present report focuses primarily on direct effects ME on carcinoma cells per se, our data also suggests that reactivation of tumor stroma (Figure 5 and Figure 6) could contribute to cancer-promoting action of this condition.

Another limitation of our study is that the TLR4 signaling mechanisms (including downstream components of TLR4 signaling cascade, e.g., MyD88, TRIF) involved in ME-associated BC progression remain insufficiently explored. Moreover, while TLR4 is currently the most extensively explored LPS receptor, emerging evidence indicate that LPS can interact with multiple other receptors and signaling pathways [82]. Thus, we cannot exclude the possibility that BC-promoting effects of ME observed here involve activation of additional targets, beyond TLR4. Further investigation is warranted in order to fully dissect the intricate network of molecular/cellular events underlying ME-associated BC. Nonetheless, our observations add a new level of complexity to the understanding of how metabolic changes influence BC behavior, highlighting ME as a potential link between obesity and breast tumorigenesis.

## Data Availability

All data generated or analyzed during this study are included in this article.

## Supplementary Materials

The following supporting information can be downloaded at: https://www.mdpi.com/article/10.3390/biomedicines13081868/s1, Figure S1. Expression of TLR4 by BC cell lines. (**A**). Human (MDA-MB-231; MCF7) and mouse (E0771) breast carcinoma cells were stained with anti TLR4 antibody (yellow). Cell nuclei were counterstained with Dapi (blue). Original magnification ×200. Scale bars: 20 μm. (**B**). Levels of TLR4 mRNA expression in human (left panel) and mouse (right panel) BC cell lines were analyzed by qRT-PCR and compared to the levels expressed by human promonocytic cell line THP-1 (right panel) and mouse primary macrophages (MAC, left panel) were used as positive controls. Data are the mean ± SD. Student’s *t*-test * *p* < 0.03; Figure S2. Kaplan–Meier plot of overall survival (OS) of BC patients with high (red line) vs. low (blue line) TLR4 expression. Plot was created using TCGA Breast Cancer (BRCA) dataset of 1247 BC patients from UCSF Xena Functional Genomics portal (https://xena.ucsc.edu); Figure S3. Accelerated growth of E0771 orthotopic tumors in mice with diet-induced obesity accompanied by ME. (**A**,**B**). Female C57BL/6J mice (8–10 week old) were fed high-fat diet (HFD, Teklad TD.06414, 60% of total calories from fat, black bars), or control diet (CD, Teklad 2018S, grey bars) for 15 consecutive weeks. (**A**). By the end of experimental week 12, when HFD-fed (but not CD-fed) animals become obese (**A**) and display ME (2twofold increase in plasma LPS levels as compared to CD-fed mice [13,49]), E0771 cells were injected orthotopically into fourth left mammary fat pads of both HFD-fed and CD- fed mice (5 × 10^5^ cells per mouse, red arrowhead), as described in [67]. (**B**). E0771 tumor growth was monitored until the end of experimental week 15. Note a marked increase in E0771 tumor volume in HFD-fed mice. Student’s *t*-test * *p* < 0.02, ** *p* < 0.004; Figure S4. Activation of STAT-3 and AKT signaling pathways in BC cells under ME conditions in vitro. (**A**–**C**). E0771 (**A**,**B**) and MCF-7 (**C**) cells were serum-starved overnight and then either remained untreated (Cont) or stimulated with LPS (0.1 ng/mL, ME). At indicated time points, cells were harvested, and cell lysates containing equivalent amounts of total protein were immunoblotted using antibodies specific for phospho-STAT3 (pSTAT3), total STAT3, phospho-AKT (pAKT), or total AKT. Band intensity was quantified using ImageJ software, and the intensity ratio (phospho/total) was calculated. Student’s *t*-test * *p* < 0.05; Figure S5. Effect of ME conditions on migration and invasion of E0771 and MCF-7 cells under in vitro. Migration (**left**) and invasion (**middle**) properties of murine E0771 (**A**) and human MCF-7 (**B**) BC cells were assessed in the absence or presence of 0.1 ng/mL LPS. Briefly, 0.6 × 10^5^ cells/mL (for migration) and 1 × 10^6^ cells/mL (for invasion) were seeded on polycarbonate membrane inserts with 8 μm pore size. CytoSelect 24-Well Cell Fluorometric Migration and Chemicon Cell Invasion assays were performed according the manufacturer’s instructions. (**Right**): ME conditions render E0771 (**A**) and human MCF-7 (**B**) cells resistant to Doxorubicin. The cells were plated on 96-well plates (in pentaplicates) and cultured either alone (control, empty bar) or in the presence of Doxorubicin (Doxo, 0.25 μM, striped bar) for 72 h. To some wells, LPS was added 3 h prior to Doxo to mimic ME conditions (ME + Doxo, dotted bar). Cell growth was analyzed by MTS Cell Proliferation Assay. Data are the mean ± SD; Student’s *t*-test * *p* < 0.05; ** *p* < 0.003; ns: statistically non-significant; Figure S6. Chronic experimental metabolic endotoxemia accelerates growth of E0771 orthotopic tumors in vivo**.** C57BL/6J mice were implanted sc. with the Alzet osmotic mini-pumps filled with either saline (Control) or LPS (to infuse 300 mg per kg per day, resulting in plasma LPS concentrations corresponding to metabolic endotoxemia, ME), as described in Methods. Three days after pump implantation syngeneic E0771 BC cells were injected orthotopically into fourth left mammary fat pads of all mice (5 × 10^5^ cells per mouse). Bar graph represents E0771 tumor volume at day 15 post injection. Student’s *t*-test * *p* < 0.02; Table S1. List of primers used in the study.

## References

[1] AACR Cancer Progress Report 2022. https://cancerprogressreport.aacr.org/progress/.

[2] Pearson-Stuttard J., Zhou B., Kontis V., Bentham J., Gunter M.J., Ezzati M. (2018). Worldwide burden of cancer attributable to diabetes and high body-mass index: A comparative risk assessment. Lancet Diabetes Endocrinol..

[3] Lee K., Kruper L., Dieli-Conwright C.M., Mortimer J.E. (2019). The Impact of Obesity on Breast Cancer Diagnosis and Treatment. Curr. Oncol. Rep..

[4] Sahin S., Erdem G.U., Karatas F., Aytekin A., Sever A.R., Ozisik Y., Altundag K. (2017). The association between body mass index and immunohistochemical subtypes in breast cancer. Breast.

[5] Pierobon M., Frankenfeld C.L. (2013). Obesity as a risk factor for triple-negative breast cancers: A systematic review and meta-analysis. Breast Cancer Res. Treat..

[6] Hillers-Ziemer L.E., Kuziel G., Williams A.E., Moore B.N., Arendt L.M. (2022). Breast cancer microenvironment and obesity: Challenges for therapy. Cancer Metastasis Rev..

[7] Chen L., Cook L.S., Tang M.T., Porter P.L., Hill D.A., Wiggins C.L., Li C.I. (2016). Body mass index and risk of luminal, HER2-overexpressing, and triple negative breast cancer. Breast Cancer Res. Treat..

[8] Fuke N., Nagata N., Suganuma H., Ota T. (2019). Regulation of Gut Microbiota and Metabolic Endotoxemia with Dietary Factors. Nutrients.

[9] Gomes J.M.G., Costa J.A., Alfenas R.C.G. (2017). Metabolic endotoxemia and diabetes mellitus: A systematic review. Metabolism.

[10] Omran F., Murphy A.M., Younis A.Z., Kyrou I., Vrbikova J., Hainer V., Sramkova P., Fried M., Ball G., Tripathi G. (2023). The impact of metabolic endotoxaemia on the browning process in human adipocytes. BMC Med..

[11] Amar J., Burcelin R., Ruidavets J.B., Cani P.D., Fauvel J., Alessi M.C., Chamontin B., Ferrieres J. (2008). Energy intake is associated with endotoxemia in apparently healthy men. Am. J. Clin. Nutr..

[12] Boutagy N.E., McMillan R.P., Frisard M.I., Hulver M.W. (2016). Metabolic endotoxemia with obesity: Is it real and is it relevant?. Biochimie.

[13] Cani P.D., Amar J., Iglesias M.A., Poggi M., Knauf C., Bastelica D., Neyrinck A.M., Fava F., Tuohy K.M., Chabo C. (2007). Metabolic endotoxemia initiates obesity and insulin resistance. Diabetes.

[14] Cao J., Peng J., An H., He Q., Boronina T., Guo S., White M.F., Cole P.A., He L. (2017). Endotoxemia-mediated activation of acetyltransferase P300 impairs insulin signaling in obesity. Nat. Commun..

[15] Troseid M., Nestvold T.K., Rudi K., Thoresen H., Nielsen E.W., Lappegard K.T. (2013). Plasma lipopolysaccharide is closely associated with glycemic control and abdominal obesity: Evidence from bariatric surgery. Diabetes Care.

[16] Cani P.D., Bibiloni R., Knauf C., Waget A., Neyrinck A.M., Delzenne N.M., Burcelin R. (2008). Changes in gut microbiota control metabolic endotoxemia-induced inflammation in high-fat diet-induced obesity and diabetes in mice. Diabetes.

[17] Thaiss C.A., Levy M., Grosheva I., Zheng D., Soffer E., Blacher E., Braverman S., Tengeler A.C., Barak O., Elazar M. (2018). Hyperglycemia drives intestinal barrier dysfunction and risk for enteric infection. Science.

[18] Neal M.D., Leaphart C., Levy R., Prince J., Billiar T.R., Watkins S., Li J., Cetin S., Ford H., Schreiber A. (2006). Enterocyte TLR4 mediates phagocytosis and translocation of bacteria across the intestinal barrier. J. Immunol..

[19] Vreugdenhil A.C., Rousseau C.H., Hartung T., Greve J.W., van ‘t Veer C., Buurman W.A. (2003). Lipopolysaccharide (LPS)-binding protein mediates LPS detoxification by chylomicrons. J. Immunol..

[20] Fitzgerald K.A., Kagan J.C. (2020). Toll-like Receptors and the Control of Immunity. Cell.

[21] Bhatelia K., Singh K., Singh R. (2014). TLRs: Linking inflammation and breast cancer. Cell Signal.

[22] Haricharan S., Brown P. (2015). TLR4 has a TP53-dependent dual role in regulating breast cancer cell growth. Proc. Natl. Acad. Sci. USA.

[23] Mai C.W., Kang Y.B., Pichika M.R. (2013). Should a Toll-like receptor 4 (TLR-4) agonist or antagonist be designed to treat cancer? TLR-4: Its expression and effects in the ten most common cancers. Onco. Targets Ther..

[24] Yang H., Wang B., Wang T., Xu L., He C., Wen H., Yan J., Su H., Zhu X. (2014). Toll-like receptor 4 prompts human breast cancer cells invasiveness via lipopolysaccharide stimulation and is overexpressed in patients with lymph node metastasis. PLoS ONE.

[25] Wang X., Yu X., Wang Q., Lu Y., Chen H. (2017). Expression and clinical significance of SATB1 and TLR4 in breast cancer. Oncol. Lett..

[26] Rakoff-Nahoum S., Medzhitov R. (2009). Toll-like receptors and cancer. Nat. Rev. Cancer.

[27] Dapito D.H., Mencin A., Gwak G.Y., Pradere J.P., Jang M.K., Mederacke I., Caviglia J.M., Khiabanian H., Adeyemi A., Bataller R. (2012). Promotion of hepatocellular carcinoma by the intestinal microbiota and TLR4. Cancer Cell.

[28] Kundu S.D., Lee C., Billips B.K., Habermacher G.M., Zhang Q., Liu V., Wong L.Y., Klumpp D.J., Thumbikat P. (2008). The toll-like receptor pathway: A novel mechanism of infection-induced carcinogenesis of prostate epithelial cells. Prostate.

[29] He Y., Ou Z., Chen X., Zu X., Liu L., Li Y., Cao Z., Chen M., Chen Z., Chen H. (2016). LPS/TLR4 Signaling Enhances TGF-beta Response Through Downregulating BAMBI During Prostatic Hyperplasia. Sci. Rep..

[30] (2017). Alzet Technical Information Manual. https://www.alzet.com/products/alzet_pumps/performance/.

[31] Fink M.P. (2014). Animal models of sepsis. Virulence.

[32] Brown G.C. (2019). The endotoxin hypothesis of neurodegeneration. J. Neuroinflammation.

[33] Maitra U., Deng H., Glaros T., Baker B., Capelluto D.G., Li Z., Li L. (2012). Molecular mechanisms responsible for the selective and low-grade induction of proinflammatory mediators in murine macrophages by lipopolysaccharide. J. Immunol..

[34] Chen K., Satlof L., Stoffels G., Kothapalli U., Ziluck N., Lema M., Poretsky L., Avtanski D. (2020). Cytokine secretion in breast cancer cells—MILLIPLEX assay data. Data Brief.

[35] Trinchieri G. (2012). Cancer and inflammation: An old intuition with rapidly evolving new concepts. Annu. Rev. Immunol..

[36] Qian B.Z., Li J., Zhang H., Kitamura T., Zhang J., Campion L.R., Kaiser E.A., Snyder L.A., Pollard J.W. (2011). CCL2 recruits inflammatory monocytes to facilitate breast-tumour metastasis. Nature.

[37] Todorovic-Rakovic N., Milovanovic J. (2013). Interleukin-8 in breast cancer progression. J. Interferon Cytokine Res..

[38] Ma Y., Ren Y., Dai Z.J., Wu C.J., Ji Y.H., Xu J. (2017). IL-6, IL-8 and TNF-alpha levels correlate with disease stage in breast cancer patients. Adv. Clin. Exp. Med..

[39] Hartman Z.C., Poage G.M., den Hollander P., Tsimelzon A., Hill J., Panupinthu N., Zhang Y., Mazumdar A., Hilsenbeck S.G., Mills G.B. (2013). Growth of triple-negative breast cancer cells relies upon coordinate autocrine expression of the proinflammatory cytokines IL-6 and IL-8. Cancer Res..

[40] Manohar S.M. (2024). At the Crossroads of TNF alpha Signaling and Cancer. Curr. Mol. Pharmacol..

[41] Karati D., Kumar D. (2024). Molecular Insight into the Apoptotic Mechanism of Cancer Cells: An Explicative Review. Curr. Mol. Pharmacol..

[42] Arendt L.M., McCready J., Keller P.J., Baker D.D., Naber S.P., Seewaldt V., Kuperwasser C. (2013). Obesity promotes breast cancer by CCL2-mediated macrophage recruitment and angiogenesis. Cancer Res..

[43] Incio J., Ligibel J.A., McManus D.T., Suboj P., Jung K., Kawaguchi K., Pinter M., Babykutty S., Chin S.M., Vardam T.D. (2018). Obesity promotes resistance to anti-VEGF therapy in breast cancer by up-regulating IL-6 and potentially FGF-2. Sci Transl. Med..

[44] Cowen S., McLaughlin S.L., Hobbs G., Coad J., Martin K.H., Olfert I.M., Vona-Davis L. (2015). High-Fat, High-Calorie Diet Enhances Mammary Carcinogenesis and Local Inflammation in MMTV-PyMT Mouse Model of Breast Cancer. Cancers.

[45] Iyengar N.M., Gucalp A., Dannenberg A.J., Hudis C.A. (2016). Obesity and Cancer Mechanisms: Tumor Microenvironment and Inflammation. J. Clin. Oncol..

[46] Tang J., Zhou B., Scott M.J., Chen L., Lai D., Fan E.K., Li Y., Wu Q., Billiar T.R., Wilson M.A. (2020). EGFR signaling augments TLR4 cell surface expression and function in macrophages via regulation of Rab5a activation. Protein Cell.

[47] Wang P., Han X., Mo B., Huang G., Wang C. (2017). LPS enhances TLR4 expression and IFN-gamma production via the TLR4/IRAK/NF-kappaB signaling pathway in rat pulmonary arterial smooth muscle cells. Mol. Med. Rep..

[48] Manilla V., Di Tommaso N., Santopaolo F., Gasbarrini A., Ponziani F.R. (2023). Endotoxemia and Gastrointestinal Cancers: Insight into the Mechanisms Underlying a Dangerous Relationship. Microorganisms.

[49] Naugler W.E., Karin M. (2008). NF-kappaB and cancer-identifying targets and mechanisms. Curr. Opin. Genet. Dev..

[50] Greenhill C.J., Rose-John S., Lissilaa R., Ferlin W., Ernst M., Hertzog P.J., Mansell A., Jenkins B.J. (2011). IL-6 trans-signaling modulates TLR4-dependent inflammatory responses via STAT3. J. Immunol..

[51] Li Y., Tang T., Sun Y., Chen G., Yuan X., Cai D. (2025). The role of TLR-4 in chemoresistance of cancer. Discov. Oncol..

[52] Guenther M., Gil L., Surendran S.A., Palm M.A., Heinemann V., von Bergwelt-Baildon M., Mayerle J., Engel J., Werner J., Boeck S. (2022). Bacterial Lipopolysaccharide as a Negative Predictor of Adjuvant Gemcitabine Efficacy in Pancreatic Cancer. JNCI Cancer Spectr..

[53] Yin H., Pu N., Chen Q., Zhang J., Zhao G., Xu X., Wang D., Kuang T., Jin D., Lou W. (2021). Gut-derived lipopolysaccharide remodels tumoral microenvironment and synergizes with PD-L1 checkpoint blockade via TLR4/MyD88/AKT/NF-kappaB pathway in pancreatic cancer. Cell Death Dis..

[54] Hu J., Li G., He X., Gao X., Pan D., Dong X., Huang W., Qiu F., Chen L.F., Hu X. (2024). Brd4 modulates metabolic endotoxemia-induced inflammation by regulating colonic macrophage infiltration in high-fat diet-fed mice. Commun. Biol..

[55] Farmer P., Bonnefoi H., Anderle P., Cameron D., Wirapati P., Becette V., Andre S., Piccart M., Campone M., Brain E. (2009). A stroma-related gene signature predicts resistance to neoadjuvant chemotherapy in breast cancer. Nat. Med..

[56] Kramer C.J.H., Vangangelt K.M.H., van Pelt G.W., Dekker T.J.A., Tollenaar R., Mesker W.E. (2019). The prognostic value of tumour-stroma ratio in primary breast cancer with special attention to triple-negative tumours: A review. Breast Cancer Res. Treat..

[57] Mao Y., Keller E.T., Garfield D.H., Shen K., Wang J. (2013). Stromal cells in tumor microenvironment and breast cancer. Cancer Metastasis Rev..

[58] Bhattacharyya S., Wang W., Qin W., Cheng K., Coulup S., Chavez S., Jiang S., Raparia K., De Almeida L.M.V., Stehlik C. (2018). TLR4-dependent fibroblast activation drives persistent organ fibrosis in skin and lung. JCI Insight.

[59] Bhattacharyya S., Midwood K.S., Yin H., Varga J. (2017). Toll-Like Receptor-4 Signaling Drives Persistent Fibroblast Activation and Prevents Fibrosis Resolution in Scleroderma. Adv. Wound Care (New Rochelle).

[60] Apte R.N. (1995). Mechanisms of cytokine production by fibroblasts-implications for normal connective tissue homeostasis and pathological conditions. Folia Microbiol. (Praha).

[61] Hersoug L.G., Moller P., Loft S. (2018). Role of microbiota-derived lipopolysaccharide in adipose tissue inflammation, adipocyte size and pyroptosis during obesity. Nutr. Res. Rev..

[62] Nahmias-Blank D., Maimon O., Meirovitz A., Sheva K., Peretz-Yablonski T., Elkin M. (2023). Excess body weight and postmenopausal breast cancer: Emerging molecular mechanisms and perspectives. Semin. Cancer. Biol..

[63] Roy S., Trinchieri G. (2017). Microbiota: A key orchestrator of cancer therapy. Nat. Rev. Cancer..

[64] Gao Z., Jiang A., Li Z., Zhu L., Mou W., Shen W., Luo P., Tang B., Zhang J., Lin A. (2025). Heterogeneity of Intratumoral Microbiota Within the Tumor Microenvironment and Relationship to Tumor Development. Med. Res..

[65] Kang L., Chen H., He C., Li J. (2025). Effects of gut microbiota in breast cancer. Front. Oncol..

[66] Arnone A.A., Ansley K., Heeke A.L., Howard-McNatt M., Cook K.L. (2025). Gut microbiota interact with breast cancer therapeutics to modulate efficacy. EMBO Mol. Med..

[67] Abdelqader E.M., Mahmoud W.S., Gebreel H.M., Kamel M.M., Abu-Elghait M. (2025). Correlation between gut microbiota dysbiosis, metabolic syndrome and breast cancer. Sci. Rep..

[68] Yang J., Tan Q., Fu Q., Zhou Y., Hu Y., Tang S., Zhou Y., Zhang J., Qiu J., Lv Q. (2017). Gastrointestinal microbiome and breast cancer: Correlations, mechanisms and potential clinical implications. Breast Cancer.

[69] Eslami S.Z., Majidzadeh A.K., Halvaei S., Babapirali F., Esmaeili R. (2020). Microbiome and Breast Cancer: New Role for an Ancient Population. Front. Oncol..

[70] Xuan C., Shamonki J.M., Chung A., Dinome M.L., Chung M., Sieling P.A., Lee D.J. (2014). Microbial dysbiosis is associated with human breast cancer. PLoS ONE.

[71] Faraj T.A., McLaughlin C.L., Erridge C. (2017). Host defenses against metabolic endotoxaemia and their impact on lipopolysaccharide detection. Int. Rev. Immunol..

[72] Violi F., Cammisotto V., Bartimoccia S., Pignatelli P., Carnevale R., Nocella C. (2023). Gut-derived low-grade endotoxaemia, atherothrombosis and cardiovascular disease. Nat. Rev. Cardiol..

[73] Soppert J., Brandt E.F., Heussen N.M., Barzakova E., Blank L.M., Kuepfer L., Hornef M.W., Trebicka J., Jankowski J., Berres M.L. (2022). Blood Endotoxin Levels as Biomarker of Nonalcoholic Fatty Liver Disease: A Systematic Review and Meta-analysis. Clin. Gastroenterol. Hepatol..

[74] Eibl G., Rozengurt E. (2021). Obesity and Pancreatic Cancer: Insight into Mechanisms. Cancers.

[75] Bhardwaj P., Au C.C., Benito-Martin A., Ladumor H., Oshchepkova S., Moges R., Brown K.A. (2019). Estrogens and breast cancer: Mechanisms involved in obesity-related development, growth and progression. J. Steroid Biochem. Mol. Biol..

[76] Argyrakopoulou G., Dalamaga M., Spyrou N., Kokkinos A. (2021). Gender Differences in Obesity-Related Cancers. Curr. Obes. Rep..

[77] Bonsang-Kitzis H., Chaltier L., Belin L., Savignoni A., Rouzier R., Sablin M.P., Lerebours F., Bidard F.C., Cottu P., Sastre-Garau X. (2015). Beyond Axillary Lymph Node Metastasis, BMI and Menopausal Status Are Prognostic Determinants for Triple-Negative Breast Cancer Treated by Neoadjuvant Chemotherapy. PLoS ONE.

[78] Pastille E., Fassnacht T., Adamczyk A., Ngo Thi Phuong N., Buer J., Westendorf A.M. (2021). Inhibition of TLR4 Signaling Impedes Tumor Growth in Colitis-Associated Colon Cancer. Front. Immunol..

[79] Yu L., Wang L., Chen S. (2010). Endogenous toll-like receptor ligands and their biological significance. J. Cell Mol. Med..

[80] Hermano E., Goldberg R., Rubinstein A.M., Sonnenblick A., Maly B., Nahmias D., Li J.P., Bakker M.A.H., van der Vlag J., Vlodavsky I. (2019). Heparanase Accelerates Obesity-Associated Breast Cancer Progression. Cancer Res..

[81] Nahmias Blank D., Hermano E., Sonnenblick A., Maimon O., Rubinstein A.M., Drai E., Maly B., Vlodavsky I., Popovtzer A., Peretz T. (2022). Macrophages Upregulate Estrogen Receptor Expression in the Model of Obesity-Associated Breast Carcinoma. Cells.

[82] De Chiara S., De Simone Carone L., Cirella R., Andretta E., Silipo A., Molinaro A., Mercogliano M., Di Lorenzo F. (2025). Beyond the Toll-Like Receptor 4. Structure-Dependent Lipopolysaccharide Recognition Systems: How far are we?. ChemMedChem.

