# Supplementary material for: Unraveling the Role of Metabolic Endotoxemia in Accelerating Breast Tumor Progression
Source: Biomedicines. 2025 Jul 31;13(8):1868. doi: 10.3390/biomedicines13081868 (PMC12383393; doi:10.3390/biomedicines13081868)
Supplement: Supplementary file 1 [file biomedicines-13-01868-s001.zip › biomedicines-3726824-supplementary.pdf]

Table S1. List of primers used in the study.

Primer name	Primer direction	Sequence 5′-3′
mouse IL-6	Forward	AGC CAG AGT CCT TCA GAG AGA TAC
	Reverse	GCC ACT CCT TCT GTG ACT CC
mouse TNFα	Forward	GCC TCT TCT CAT TCC TGC TTG
	Reverse	CTG ATG AGA GGG AGG CCA TT
mouse IL-10	Forward	TCC CCT GTG AAA ATA AGA GCA A
	Reverse	ACC TTG GTC TTG GAG CTT ATT AA
mouse IL-1β	Forward	CAA CCA ACA AGT GAT ATT CTC CAT G
	Reverse	GAT CCA CAC TCT CCA GCT GCA
mouse CCL2	Forward	GCA TCC ACG TGT TGG CTC A
	Reverse	AGC CTA CTC ATT GGG ATC ATC TTG
mouse TLR4	Forward	ATG CAT GGA TCA GAA ACT CAG CAA
	Reverse	AAA CTT CCT GGG GAA AAA CTC TGG
mouse HPRT	Forward	GTC GTG ATT AGC GAT GAT GAA
	Reverse	CTC CCA TCT CCT TCA TGA CAT C
human IL-8	Forward	ACT GAG AGT GAT TGA GAG TGG AC
	Reverse	AAC CCT CTG CAC CCA GTT TTC
human IL-6	Forward	GGC ACT GGC AGA AAA C
	Reverse	GCA AGT CTC CTC ATT G
human TNFα	Forward	CTG CCC CAA TCC CTT T
	Reverse	CCC AAT TCT CTT TTT G
human TLR4	Forward	ACC AAG AAC CTG GAC CTG AG
	Reverse	TCT GGA TGG GGT TTC CTG TC
human CCL2	Forward	CAG CCA GAT GCA ATC AAT GCC
	Reverse	TGG AAT CCT GAA CCC ACT TCT
human HPRT	Forward	GCT ATA AAT TCT TTG CTG ACC TGC T
	Reverse	ATT ACT TTT ATG TCC CCT GTT GAC TG

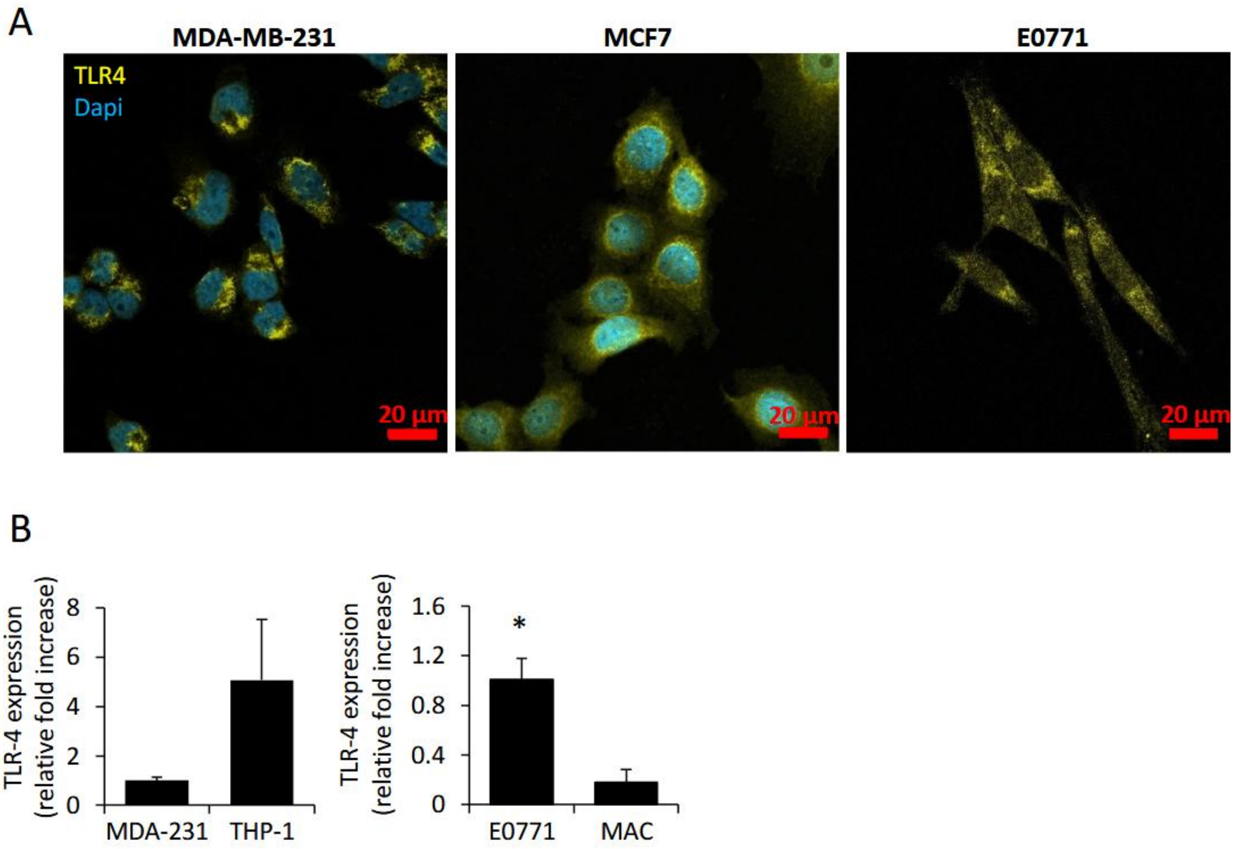

Figure S1. Expression of TLR4 by BC cell lines. **A**. Human (MDA-MB-231; MCF7) and mouse (E0771) breast carcinoma cells were stained with anti TLR4 antibody (yellow). Cell nuclei were counterstained with Dapi (blue). Original magnification ×200. Scale bars: 20 μm. **B**. Levels of TLR4 mRNA expression in human (left panel) and mouse (right panel) BC cell lines were analyzed by qRT-PCR and compared to the levels expressed by human promonocytic cell line THP-1 (right panel) and mouse primary macrophages (MAC, left panel) were used as positive controls. Data are the mean ± SD. Student's t-test $*p < 0.03$.

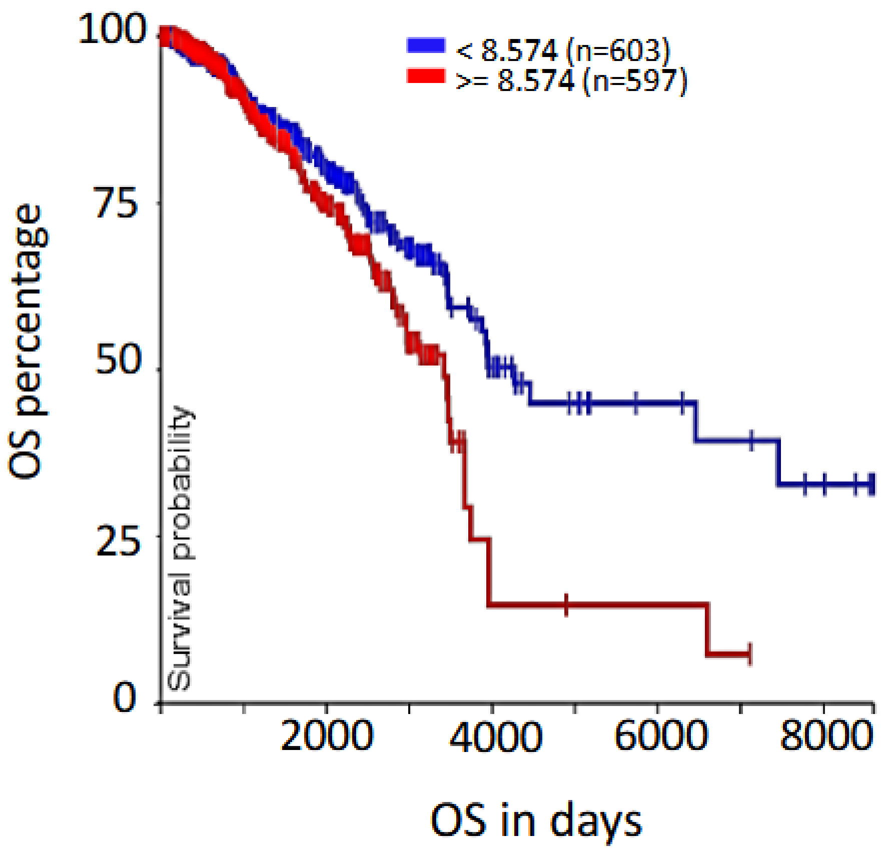

Figure S2. Kaplan–Meier plot of overall survival (OS) of BC patients with high (red line) vs. low (blue line) TLR4 expression. Plot was created using TCGA Breast Cancer (BRCA) dataset of 1247 BC patients from UCSF Xena Functional Genomics portal (https://xena.ucsc.edu).

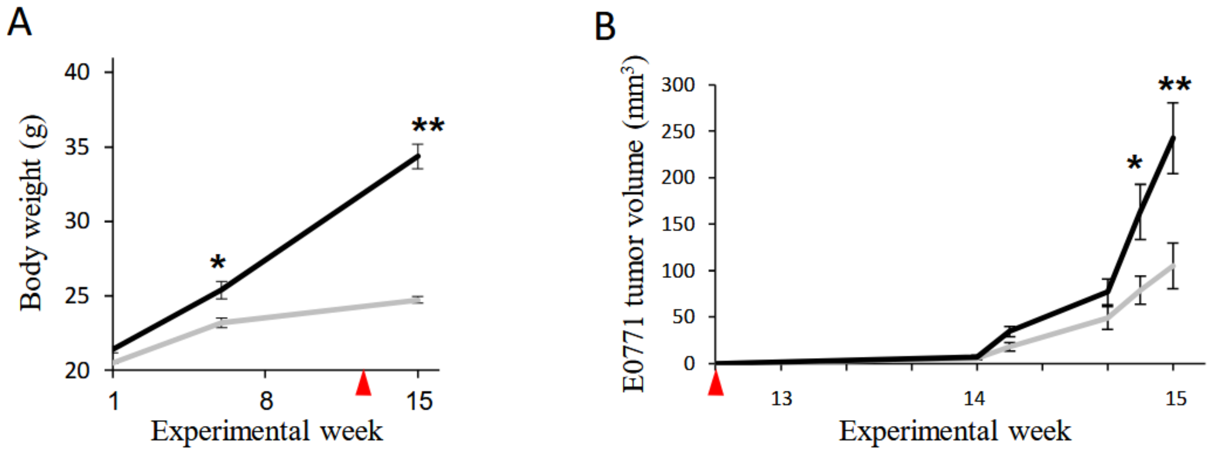

Figure S3. Accelerated growth of E0771 orthotopic tumors in mice with diet-induced obesity accompanied by ME. **A, B.** Female C57BL/6J mice (8–10 week old) were fed high-fat diet (HFD, Teklad TD.06414, 60% of total calories from fat, black bars), or control diet (CD, Teklad 2018S, grey bars) for 15 consecutive weeks. **A**. By the end of experimental week 12, when HFD-fed (but not CD-fed) animals become obese (**A**) and display ME (2twofold increase in plasma LPS levels as compared to CD-fed mice, [13,49]), E0771 cells were injected orthotopically into fourth left mammary fat pads of both HFD-fed and CD- fed mice (5×10^5 cells per mouse, red arrowhead), as described in [67]. **B**. E0771 tumor growth was monitored until the end of experimental week 15. Note a marked increase in E0771 tumor volume in HFD-fed mice. Student's t-test *$p < 0.02$, **$p < 0.004$.

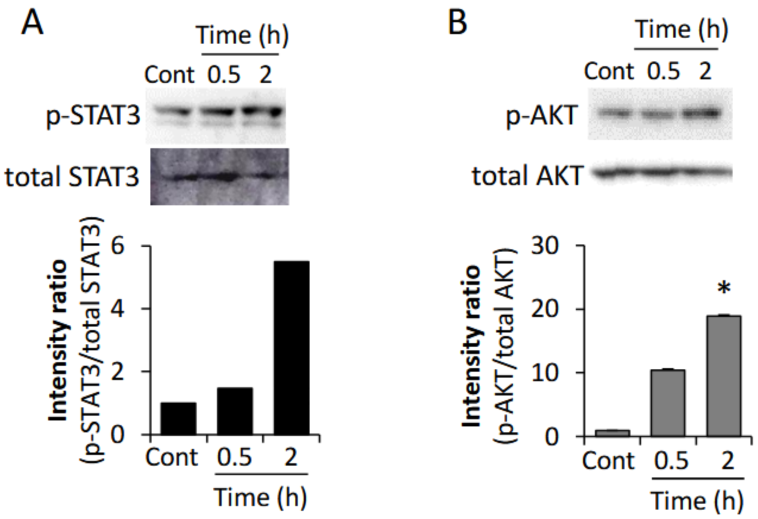

Figure S4. Activation of STAT-3 and AKT signaling pathways in BC cells under ME conditions in vitro. **A–C.** E0771 (**A, B**) and MCF-7 (**C**) cells were serum-starved overnight and then either remained untreated (Cont) or stimulated with LPS (0.1 ng/mL, ME). At indicated time points, cells were harvested, and cell lysates containing equivalent amounts of total protein were immunoblotted using antibodies specific for phospho-STAT3 (pSTAT3), total STAT3, phospho-AKT (pAKT), or total AKT. Band intensity was quantified using ImageJ software, and the intensity ratio (phospho/total) was calculated. Student's t-test *$p < 0.05$.

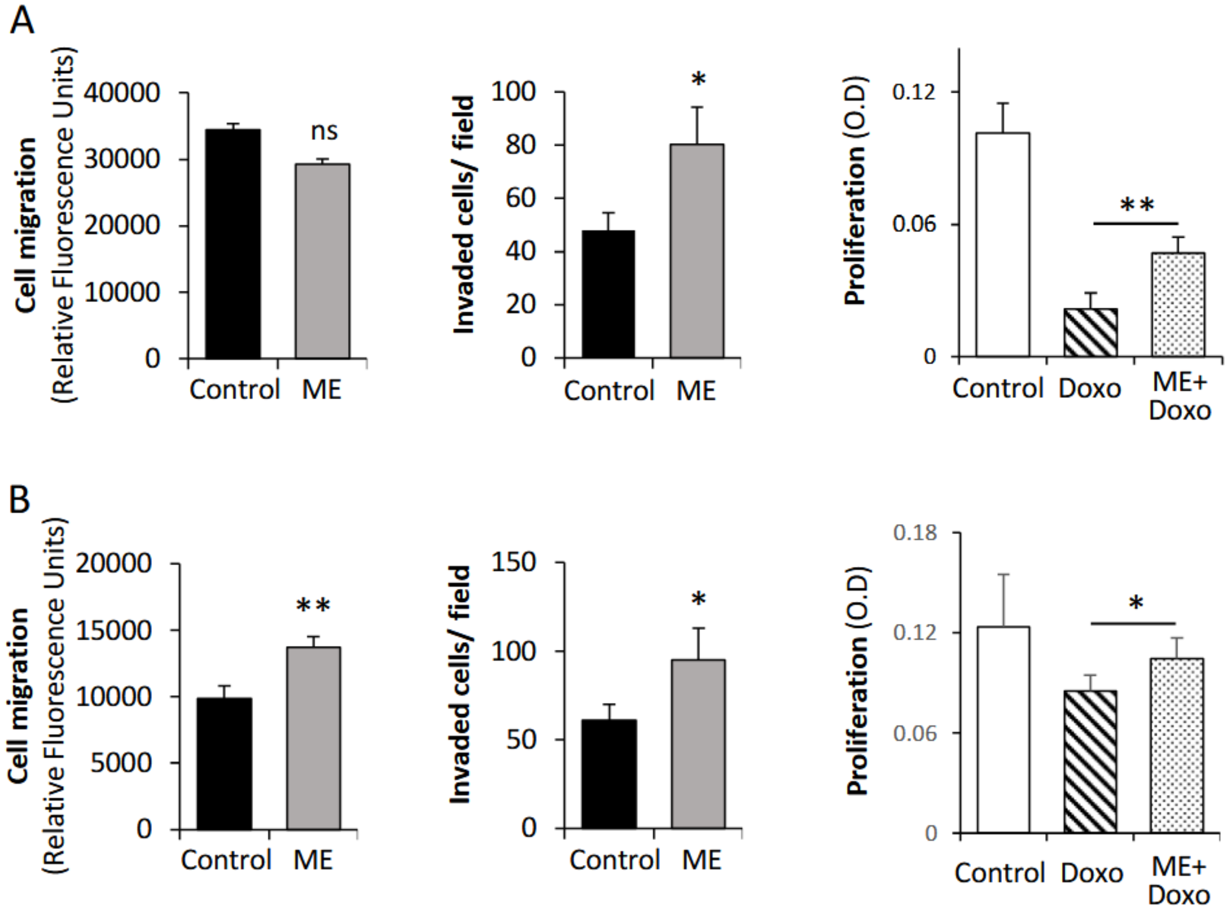

Figure S5. Effect of ME conditions on migration and invasion of E0771 and MCF-7 cells under in vitro. Migration (**left**) and invasion (**middle**) properties of murine E0771 (**A**) and human MCF-7 (**B**)

BC cells were assessed in the absence or presence of 0.1 ng/mL LPS. Briefly, 0.6×10^5 cells/mL (for migration) and 1×10^6 cells/mL (for invasion) were seeded on polycarbonate membrane inserts with 8 μm pore size. CytoSelect 24-Well Cell Fluorometric Migration and Chemicon Cell Invasion assays were performed according the manufacturer's instructions. **Right**: ME conditions render E0771 (**A**) and human MCF-7 (**B**) cells resistant to Doxorubicin. The cells were plated on 96-well plates (in pentaplicates) and cultured either alone (control, empty bar) or in the presence of Doxorubicin (Doxo, 0.25 μM, striped bar) for 72 h. To some wells, LPS was added 3 h prior to Doxo to mimic ME conditions (ME + Doxo, dotted bar). Cell growth was analyzed by MTS Cell Proliferation Assay. Data are the mean ± SD; Student's t-test $*p < 0.05$; $**p < 0.003$; ns: statistically non-significant.

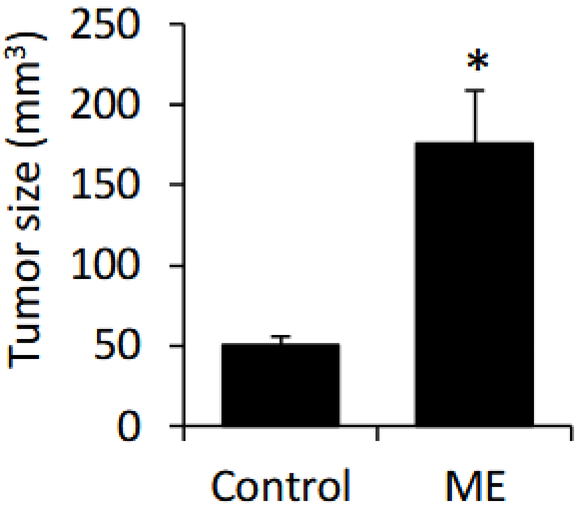

Figure S6. Chronic experimental metabolic endotoxemia accelerates growth of E0771 orthotopic tumors in vivo**.** C57BL/6J mice were implanted sc. with the Alzet osmotic mini-pumps filled with either saline (Control) or LPS (to infuse 300 mg per kg per day, resulting in plasma LPS concentrations corresponding to metabolic endotoxemia, ME), as described in Methods. Three days after pump implantation syngeneic E0771 BC cells were injected orthotopically into fourth left mammary fat pads of all mice (5×10^5 cells per mouse). Bar graph represents E0771 tumor volume at day 15 post injection. Student's t-test $*p < 0.02$.
